# Brain-derived nerve growth factor in the cochlea – a reproducibility study

**DOI:** 10.1186/s40463-020-00432-7

**Published:** 2020-06-05

**Authors:** Brian W. Blakley, Michael Seaman, Abdulrahman Alenezi

**Affiliations:** Department of Otolaryngology, University of Manitoba, Winnipeg, Manitoba R3A 1R9 Canada

**Keywords:** Hearing, Brain-derived nerve growth factor, Auditory brainstem response, Ototoxicity

## Abstract

**Objective:**

Brain-derived nerve growth factor (BDNF) plays an important role in cochlear development so it is plausible that it could restore hearing loss if delivered directly into the cochlea. We wished to confirm our previous report that a single intracochlear injection of brain-derived nerve growth factor (BDNF) was beneficial for hearing in guinea pigs. We wished to assess the reproducibility of our results and assess possible improved methods with a view to developing a clinical treatment for sensorineural hearing loss.

**Methods:**

CDDP was used to create partial hearing loss in 25 guinea pigs. After 30 days the animals underwent ABR testing and unilateral BDNF injection through the round window in one ear and saline injection into the other ear. After allowing possible effects to stabilize, thirty days later, ABR threshold testing was repeated to assess change in threshold.

**Results:**

Final ABR thresholds were 60–70 dB and were about 11 dB better in the ears treated with BDNF.

**Conclusion:**

Our original finding that Intracochlear BDNF can improve hearing in guinea pigs was confirmed, but the improvement demonstrated by the methods in this paper is too small for clinical application.

## Introduction

Brain-derived nerve growth factor (BDNF) is important for hearing. BDNF is known to play a significant role in cochlear development [1], but its role in adults is less certain [2]. Several authors have reported that BDNF protects against ototoxicity [3] or is important in spiral ganglion cell maintenance [4, 5] but a larger, real-world need is to treat chronic sensorineural hearing loss. Some papers have suggested that BDNF expression restores or preserves auditory nerve morphology, electrical brainstem responses or neurophysiology in guinea pigs after ototoxic hearing loss [6–13]. Others have suggested that BDNF application may improve outcomes in cochlear implantation [14].

Gene therapy is one consideration. Ongoing research involving promising, molecular biological approaches to hearing loss may provide treatment someday but knowledge of gene control and methods of administration of genetic modifiers is lacking. Could simple injection of BDNF to the cochlea achieve positive results? The answer to this question is unclear but it seems important to address a possible simple solution for hearing loss. After all, the research findings that relate to BDNF and hearing are based on the expression of BDNF, not control of its activity. The possibility that direct injection of BDNF into the cochlea deserves study as a possible clinical intervention for hearing loss.

There is uncertainty regarding BDNF research. Much of the BDNF research focuses on hair cell survival or in vitro findings, not actual hearing. BDNF may result in morphologic improvements but not hearing in pigeons [15]. For clinicians, the glaring deficiency in the literature seems to be the lack of evidence that hearing is actually improved. In some previous studies BDNF was applied to the round window niche, sometimes over several weeks. To us this seemed unlikely to succeed considering the blood-labyrinth barrier and the fact that BDNF is a high molecular weight substance that may not pass through the round window. In addition, many studies assess the effect of BDNF or some other agent only a few days after exposure to an ototoxic agent. This may not provide valid information if the hearing loss has not stabilized. In previous publications we reported some data hoping that our methods would be useful in restoring hearing [16, 17], but data conflict so we undertook more studies to validate and possibly improve on our previous results.

We considered some improvements on our initial design. In our previous study [16] we used intraperitoneal cisplatin (cisdiaminedichloroplatinum or CDDP) 15 mg/kg to create hearing loss, then drilled a hole through the cochlea, possibly violating the stria vascularis, to deliver 0.05 micrograms of BDNF as a single application. Six of 16 guinea pigs had to be euthanized, due to weight loss, so we were left with 11 animals. We desired a study with more surviving animals. We have found that CDDP 4 mg/kg on alternate days for 3 doses (total dose 12 mg/kg) resulted in moderate hearing loss, but minimal mortality. In addition, the “soft approach” cochlear implantation [18] led us to suspect that we would find better results if we delivered the BDNF through the round window, rather than drilling into the cochlea.

Our lab has previously explored the possibility that simply injecting the BDNF into the cochlea is associated with better hearing. If such a simple treatment were successful, the application could help millions. The purpose of this study was to assess the reproducibility and possibly confirm our previous findings. In our previous study we reported that a single intracochlear injection of BDNF resulted in auditory brainstem response (ABR) thresholds across several frequencies that were from 2 to 27 dB better than saline-injected ears [16]. We found these results encouraging and hoped that, with modification, they could be applicable clinically to humans if the techniques are effective. In a subsequent study using more traumatic techniques than herein, we found that click thresholds were not statistically different in ears treated with BDNF [17]. We felt that the differences between pure tone and click thresholds were conflicting results that must be resolved with this study.

Neurotrophins such as BDNF act by signaling through tyrosine kinases, reducing the formation of oxygen free radicals and up-regulation of apoptotic genes and mitigation of intracellular calcium (Ca^2+^) activity. BDNF acts on the TrkB receptor. Mice that lack either BDNF or the TrkB receptor have no hearing [19].

Several studies have suggested that BDNF could be useful in preservation of auditory structure or function in animals [13, 20–22] and humans [23] but the technique has not seen clinical application to date [24]. BDNF may be the most important neurotrophin for maintenance of cochlear neurons [25, 26]. In fact, the “neurotrophin factor hypothesis” posits that all age-related degeneration in the central nervous system results from deficiencies of neurotrophins [4].

In vitro, BDNF has been shown to be important BDNF seems to be the most important in the cochlea [2, 27] and for cochlear development and maintenance of cochlear histology in adult life [23, 28, 29]. BDNF is up-regulated in the organ of Corti but not auditory neurons in gerbils, suggesting that BDNF may mitigate cochlear hearing losses to a greater extent that those due to auditory nerve degeneration [30].

There is reason to suspect that the activity of BDNF on cochlear cells is not under strict genetic control and a single application of BDNF may be effective [31].

If this project is successful it appears that BDNF could be applied systemically to humans. Clinical trials conducted using daily BDNF to treat amyotrophic lateral sclerosis [32, 33], Guillian-Bare [34], and diabetic neuropathy [35] have suggested favorable outcomes for those disorders. Nausea and vomiting was the most common side effect of systemic but not intrathecal administration. Hearing was not assessed in those trials but the studies illustrate that BDNF could be safely delivered to humans. The inner ear is relatively isolated from systemic circulation by the blood-labyrinth barrier so it seems likely that systemic toxicity should be minimal with intracochlear administration. BDNF has a molecular weight of 27.5 kDa which is too large to allow BDNF to pass through the blood-labyrinth barrier. Systemic administration of BDNF would result in low concentrations and likely induce systemic side effects so a local method of drug administration seems optimal. The simplest method of introducing drugs into the inner ear locally would seem to be simple injection through the round window, plugging the needle opening after injection [36].

An effective neurotrophin should require time to achieve complete effect. Ruan et al. [37] reported that BDNF supported cochlear histology 30 days after administration of kanamycin to induce ototoxicity but not 15 days. BDNF “rescue” may be effective even if administered more than 2 weeks after inducing hearing loss [6] or longer [9].

Our findings mimic those of Radeloff and Smolders [15] who delivered BDNF over 8 weeks with osmotic micropumps but found that the insertion trauma outweighed the positive effects of the BDNF. As clinicians, we suspect that chronical implantation of micropumps would create a perilymph fistula and subsequent hearing loss and favor intermittent applications.

For this project we adopted some of the principles of “soft technique” for cochlear implantation that were intended to preserve residual hearing. Those relevant to this project these include slow, gentle, shallow injection, use of the round window rather than a cochleostomy with a drill [38], and avoiding the entry of blood into the cochlea [18, 38].

This study is our attempt to re-produce the results of our previous study using more animals, a lower, more tolerable dose of CDDP and less traumatic, round window delivery of BDNF. The protocol was approved by the University of Manitoba Animal Ethics Research Committee.

## Methods

**Fig. 1:**
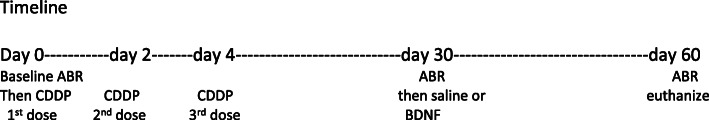
Timeline for experiments. After initial auditory brainstem response (ABR) testing the 25 guinea pigs received cisplatin (CDDP, 4 mg/kg X 3 doses on alternate days for a total of 12 mg/kg). After allowing hearing loss to stabilize for a month either BDNF or saline was injected into the round window. ABR thresholds were obtained another month after that

Our ABR protocol has been described previously [16]. Pure tone ABR threshold testing was performed under ketamine anesthesia at 3 K, 6 K, 12 K and 24 K Hz using the Intelligent Hearing Systems platform for evoked potentials. After baseline thresholds (day 0) in dB SPL were obtained, intraperitoneal CDDP (4 mg/kg X 3 doses on alternate days for a total dose of 12 mg/kg) was administered to create a partial sensorineural hearing loss. After allowing 30 days for the hearing to stabilize (day 30), ABR testing was performed again, and under the same anesthetic, the left round window in each animal was injected with 0.05 micrograms of BDNF (Sigma corp) and the same volume (0.01 cc) of saline injected into the right round window. These injections were accomplished after removing some of the tympanic membrane and part of the scutum to visualize the round window under a microscope. An opening in the round window was created by puncturing it with a sharp pick. The injections were performed by placing a finely calibrated gas chromatograph syringe through the opening in the round window into the cochlea. A small piece of fat was then used to seal the round window. Healing of the tympanic membranes occurred over 10–14 days and was verified by otoscopy. Thirty days after the injections (day 60) ABR was performed again to assess the final hearing result. The timeline is shown in Fig. 1.

ABR thresholds were entered into a database and Analysis of Variance for Repeated Measures was performed using SPSS v24 software and a significance level of *p* = 0.05 to assess the differences in threshold across the treated versus non-treated ears over the days of testing and across the four frequencies tested. Contrasts were employed to determine the significance of differences in threshold according to day number (0, 30 or 60).

## Results

**Fig. 2:**
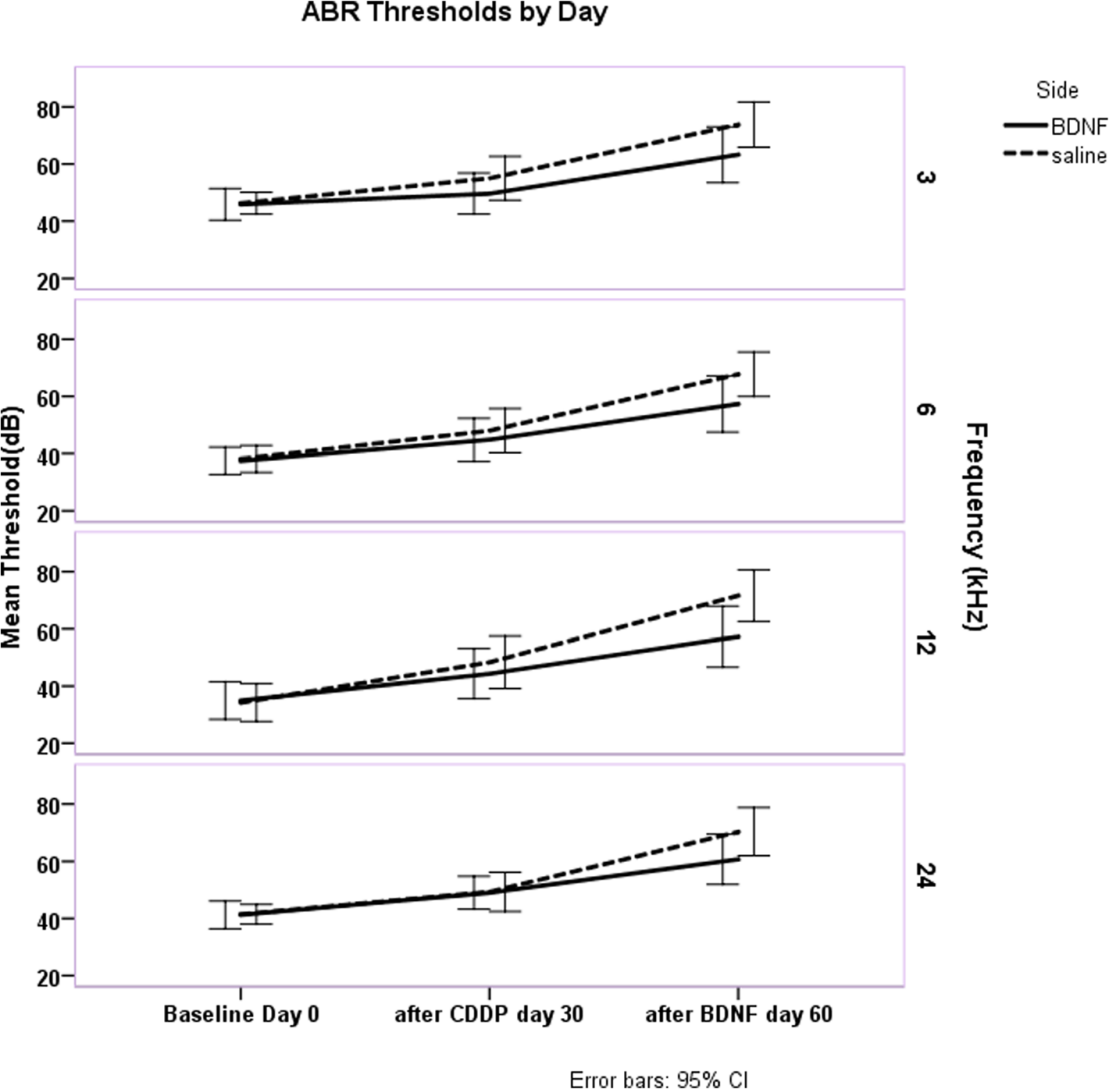
ABR thresholds by day across the four frequencies (3 K, 6 k, 12 k and 24 K Hz) tested. Day 0 is the initial day. Day 30 represents the thresholds after CDDP administration but before BDNF/saline administration and day 60 represents the thresholds after BDNF or saline administration. Although the thresholds are greater at day 60 they do not differ significantly by frequency (*p* = 0.088). thresholds differ between day 30 and day 60 in both groups. This difference in the saline group probably represents the hearing loss induced by the intracochlear injection procedure alone (21 dB) which is of concern if these methods were to be applied to humans

Figure 2 illustrates the changes in ABR threshold for the four frequencies tested at days 0 (baseline), day 30 (after CDDP), and day 60 (after BDNF/saline) rounding ABR thresholds and 95% CI results to two significant figures. Mauchly’s test of sphericity was not violated (*p* = 0.254) for ABR thresholds by day. ANOVA results indicates that differences across frequencies were not statistically significant (*p* = 0.088) so all frequencies were included together in further analysis.

**Fig. 3:**
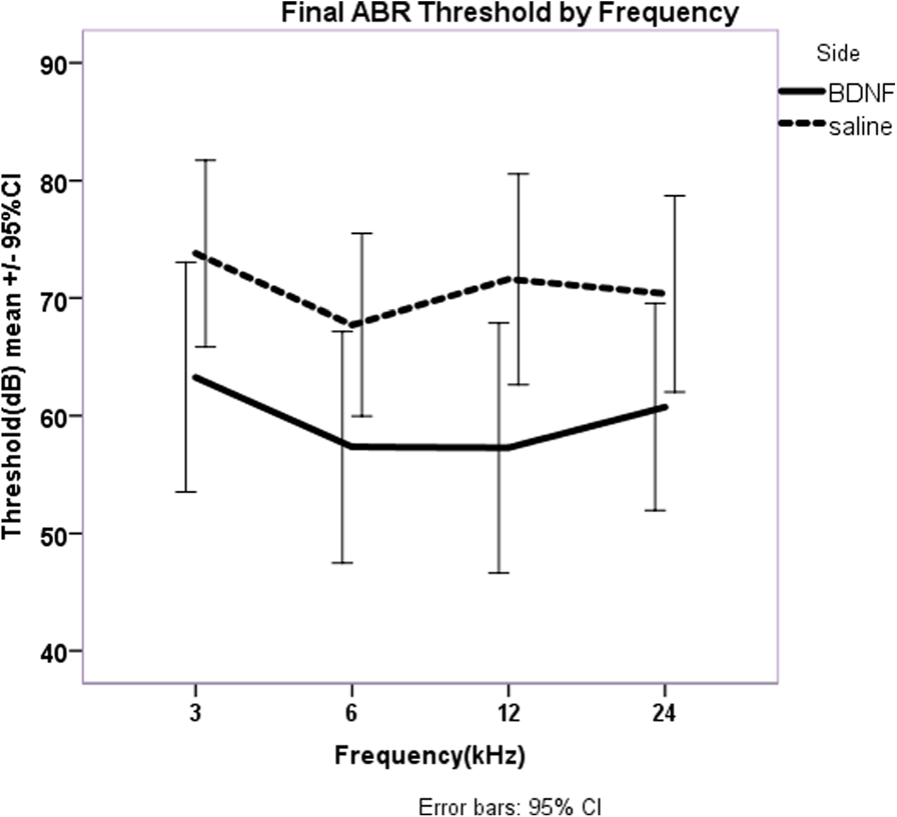
Averaged final thresholds at day 60 with all frequencies included. The mean difference in ABR thresholds for BDNF-treated ears (60 were 60+/− 23 dB) versus saline-treated ears (71 +/− 20 dB) was significant (*p* = 0.034). The mean difference was 11 dB, which is less than the increase in threshold resulting from the intracochlear injection

Threshold differences were not significantly different between the treated and non-treated ears at days 0 or 30, but at day 60 (30 days after BDNF/saline treatment) the traces diverged significantly. Figure 3 illustrates the final ABR thresholds for BDNF-treated and saline-treated ears. Statistically significant differences in ABR threshold were demonstrated between the BDNF-treated and saline-treated ears (*p* = 0.034). Across all frequencies final, rounded ABR thresholds in BDNF-treated ears on day 60 were 60+/− 23 dB (mean +/− 95%CI) in BDNF-treated ears and 71 +/− 20 dB in the saline-treated ears for a mean difference of 11 dB.

CDDP induced a mean increase in ABR threshold on both sides of 9 dB across all frequencies. Intracochlear injection increased the thresholds further by an average of 16 dB (21 dB on the saline side and 13 dB on the BDNF side).

## Discussion

Several findings deserve discussion.

First, our results indicate that intracochlear BDNF is associated with lower thresholds of about 11 dB. The results of this study are consistent with some of our previous work, indicating a small difference in threshold in ears treated with BDNF [16]. We were hoping to demonstrate that intracochlear BDNF treatment can restore hearing. While a gain of 11 dB is desirable this gain is not adequate to apply clinically. Thresholds are still far from normal. It appears that a single treatment of BDNF delivered as we have done here, will not provide treatment for sensorineural hearing loss as we had hoped. Perhaps multiple applications, a different dose or other method would show more response. We plan to pursue a multiple dose regimen in future work.

Second, the 11 dB improvement in hearing did not overcome the loss induced by the intracochlear injection, highlighting the importance of controls in research. The effect of the injection procedure is evident as the increase in threshold between 30 and 60 days in both the BDNF- and the saline-treated ears. This finding would seem to be important for any agent injected into the cochlea for the purpose of improving hearing.

Third, it is odd that the ABR thresholds did not differ by frequency as one might expect from the human literature. In this project as well as our previous work has also found that, in rodents, CDDP affects all thresholds across the range of frequencies tested. Differences between human and rodent hearing should be considered if this research is to be applied to humans. For example, rodents have excellent hearing in higher frequency ranges than humans, perhaps up to 40 kHz. We have sampled some of both ranges. Anesthetic time limits the feasibility of testing at very many frequencies.

Fourth, the CDDP did not create as much hearing loss as it has in past work in our lab [39]. Typically, we expect to induce a loss of about 25–30 dB with the CDDP-regimen used here. This illustrated the variability in animal research models. Perhaps with large hearing losses our results would be different.

Although it CDDP is commonly thought to affect higher frequencies more than lower ones this was not reflected in our data. ABR thresholds at day 30 were similar across frequencies. We have found this in other studies as well [39].

## Conclusion

Intracochlear BDNF injection can benefit hearing in guinea pigs but does not overcome the hearing loss induced by the injection itself. The methods as used in this paper are inadequate for clinical application.
